# Immune checkpoint inhibitors for multiple myeloma immunotherapy

**DOI:** 10.1186/s40164-023-00456-5

**Published:** 2023-11-28

**Authors:** Zhaoyun Liu, Xintong Xu, Hui Liu, Xianghong Zhao, Chun Yang, Rong Fu

**Affiliations:** Department of Hematology, Tianjin Medical University General Hospital, Tianjin, 300052 China

**Keywords:** Immune checkpoint, CTLA-4, TIGIT, Multiple myeloma, treatment strategy

## Abstract

Multiple myeloma (MM) is related to immune disorders, recent studys have revealed that immunotherapy can greatly benefit MM patients. Immune checkpoints can negatively modulate the immune system and are closely associated with immune escape. Immune checkpoint-related therapy has attracted much attention and research in MM. However, the efficacy of those therapies need further improvements. There need more thoughts about the immune checkpoint to translate their use in clinical work. In our review, we aggregated the currently known immune checkpoints and their corresponding ligands, further more we propose various ways of potential translation applying treatment based on immune checkpoints for MM patients.

## Introduction

Intratumoural heterogeneity that occurs during tumour evolution is the most critical cause of cancer death, treatment failure and drug resistance [1, 2]. The biological capabilities developed during tumourigenesis and progression are manifested in six main areas: maintenance of proliferative signaling, evasion of growth inhibitors, obstruction of cell death, guidance of replicative immortality, induction of angiogenesis, activation of metastasis and invasion of other sites. These features are associated with genomic instability and genetic diversity, which in turn lead to tumour complexity [3]; however, the genetic and cytological changes in the tumor provide assistance with diagnosis and treatment. Under ideal conditions, the normal immune process can be summarised as the 'cancer immune cycle’: the tissue surrounding the tumour releases inflammatory cytokines that direct the accumulation of dendritic cells. Upon arrival of the dendritic cells at the tumour, the ‘tumour antigens’ released by the tumour cells are presented to naïve T lymphocytes via the major histocompatibility complex (MHC) I. The naïve T lymphocytes are activated and they differentiate into mature T lymphocytes, which are capable of recognising and attacking the tumour and activating into effector T cells, thereby initiating and activating a response against cancer specific antigens. Finally, T cell activation influences migration towards the vasculature and the infiltrating tumour microenvironment; the T cell receptor (TCR) interacts with MHC I leading to the recognition of specific cognate antigens and their binding to cancer cells; and Fas–Fas ligands interact with each other releasing specific substances, including enzymes or perforin particles, which are cytotoxic leading to the killing of the targeted cancer cells [4, 5].

MM, a malignant plasma cell disease in which clonal plasma cells in the bone marrow proliferate and are characterized by the formation of specific monoclonal immunoglobulin bands, can cause damage to multiple organs or tissues. MM induces multiple organ damage, which typically includes anemia, renal impairment, lytic bony lesions, and hypercalcemia, referred to simply as “CRAB” [6, 7]. In MM, T cells bind to antigens displayed on cancer cells, resulting in T cell inactivation, which leads to humoral and cellular immune dysfunction and altered immune surveillance, supporting tumor progression and immune escape [8, 9].

Immune checkpoints can function as negative regulators of the immune system that prevent autoimmunity and protect tissues from attack by an overactive immune system [10]. However, tumor cells use this feature for immune evasion. Under the guidance of the cancer immune cycle, the clinical application of immune checkpoint inhibitors (ICIs) aims to interrupt the co-inhibitory pathway, facilitate the release preexisting anti-tumor immune effectors, and reset or restore dysfunctional effector T cells, thereby promoting immune-mediated elimination of tumor cells [11–13]. Immune regulation mechanisms also play an important role in hematological malignancies. Immune checkpoint inhibitor-related monoclonal antibodies have been continuously developed, and diagnostic and treatment protocols have been proposed. This article discusses and summarizes the immune checkpoints and related therapeutic strategies that have been discovered so far, with the aim of proposing new diagnostic and treatment protocols for hematological malignancies.

**Fig. 1:**
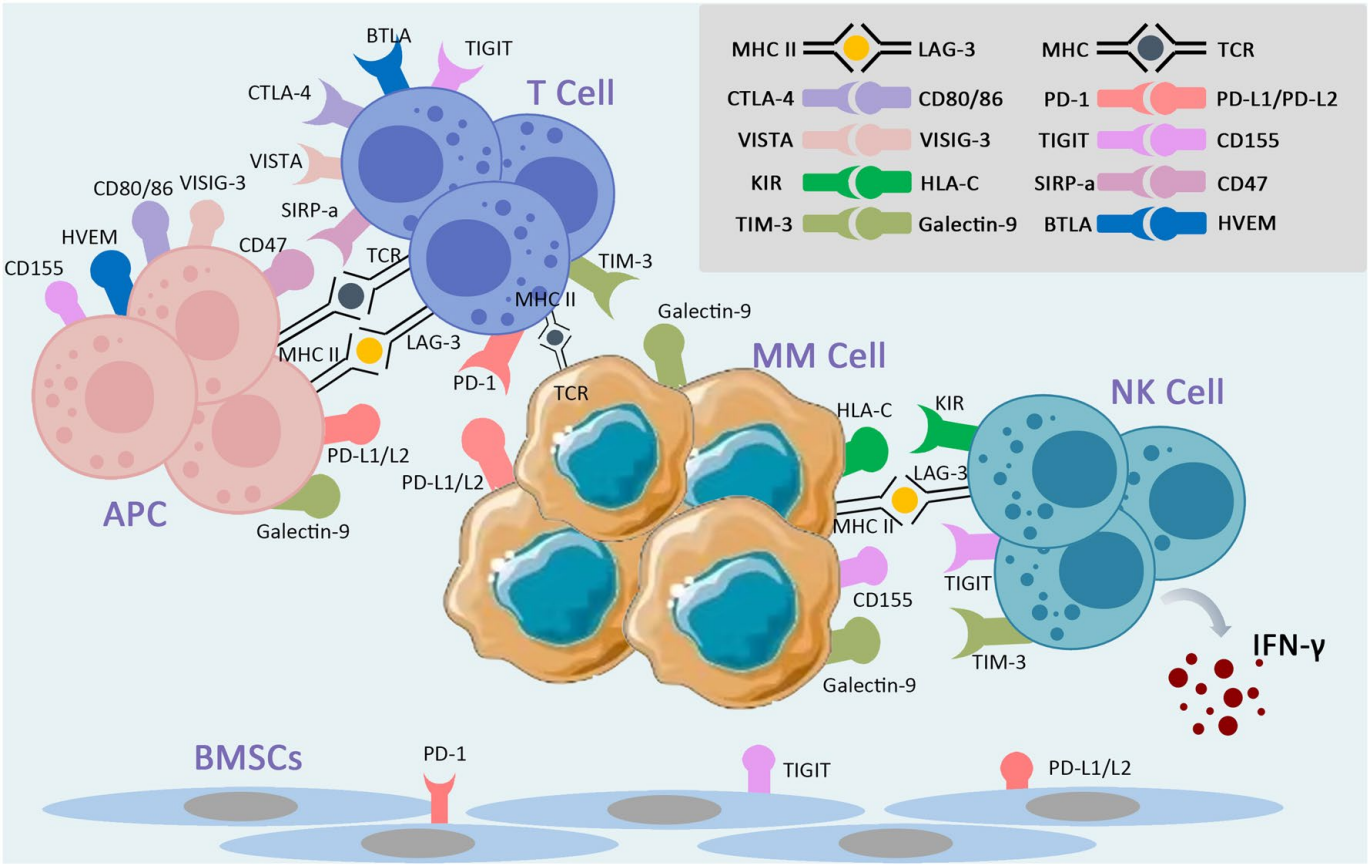
Immune checkpoints in MM

Immune checkpoints maintain self-tolerance by modulating the immune system and establish self-protection mechanisms by eliminating cells produced by an overactive immune system. However, this is also an important mechanism that leads to the growth and spread of tumor cells and drug resistance [9, 10]. After the unremitting efforts of researchers, many immune checkpoints have been discovered and have brought new hope to tumor treatment. In order to form a tumor microenvironment (TME) suitable for tumor survival, tumor cells undergo a series of preparations, and stimulating the activity of suppressive immune checkpoints is also an important part of it [14]. Studies have shown that one of the mechanisms of immune escape in MM is the upregulation of immune checkpoints, destroying the function of effector T cells [15–17]. Concurrently, changes in immune checkpoint expression are directly related to the prognosis of MM patients (Fig. 1).

## Mechanisms of immune checkpoint in multiple myeloma

**Fig. 2:**
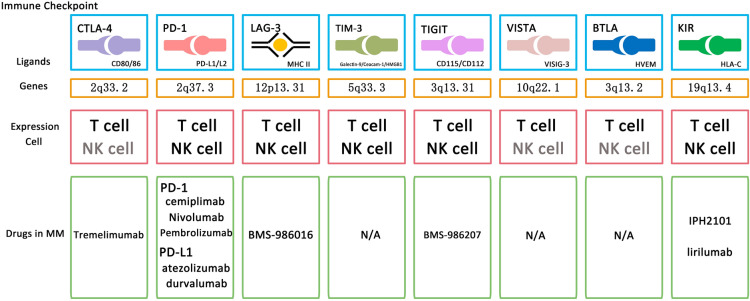
Mechanisms of immune checkpoint in multiple myeloma

With the development of scientific research, many immune checkpoints associated with MM have been discovered in recent years, and by studying their structures and related pathogenic mechanisms, new ideas for the treatment of MM have also been provided. Most immune checkpoints belong to the immunoglobulin superfamily, which consists of proteins containing one or more structural domains, among which the variable (V) and constant (C) immunoglobulin structural domains are the key structural features for their diversity and function through immunoglobulins, TCR, MHC I and MHC II [18]. The type and expression of these immune checkpoints varies from cell to cell and is highly variable in myeloma cells [19]. We introduce several common immune checkpoints in MM (Fig. 2, Table 1). In addition, we summarize the current clinical trial data for all MM immune checkpoints with reference to CliniclTrial.gov (Table 2).

**Table 1:** MM-associated immune checkpoints and their ligands, gene locations, and expressing cells

Immune checkpoint	Ligands	Genes location	Expression cells
CTLA-4	CD80/CD86	2q33.2	T Cell
PD-1	PD-L1/PD-L2	2q37.3	T Cell/NK cell
LAG-3	MHC II	12p13.31	T Cell/NK cell
TIM-3	Galectin-9/Ceacam-1/HMGB1	5q33.3	T Cell/NK cell
TIGIT	CD115/CD112	3q13.31	T Cell/NK cell
VISTA	VISIG-3	10q22.1	T Cell
BTLA	HVEM	3q13.2	T Cell
KIR	HLA-C	19q13.4	T Cell/NK cell

**Table 2:** Current clinical trail data of Checkpoint targets and agents

Target	Agent(s)	Type	ClinicalTrials.gov identifier	Trials in myeloma	Status
CTLA-4	Tremelimumab	Human	NCT02716805	Phase 1 trail in MM In combination of Durvalumab, High-dose Chemotherapy after ASCT	Terminated
PD-1	Pembrolizumab	Humanized	NCT03848845	Phase 1 trail in RRMM In Combination With GSK2857916	Active, not recruiting
			NCT02576977	Phase 3 trail in RRMM Pomalidomide and Low Dose Dexamethasone with or without Pembrolizumab	Terminated
			NCT02906332	Phase 3 trail in High-risk MM In combination of lenalidomide post ASCT	Active, not recruiting
			NCT03221634	Phase 2 trail in RRMM In Combination With Daratumumab	Withdrawn
			NCT05493618	Phase 1/2 trail in Refractory MM In Combination With Belantamab and Dexamethasone	Not yet recruiting
			NCT02579863	Phase 3 trail in NDMM Lenalidomide and Dexamethasone with or without Pembrolizumab	Terminated
			NCT03191981	Phase 1/2 trail in Refractory MM In combination with Cyclophosphamide and Lenalidomide	Withdrawn
			NCT02289222	Phase 1/2 trail in RRMM In combination with IMiD (Pomalidomide)	Terminated
			NCT05191472	Phase 2 trail in RRMM In combination with Anti-BCMA CAR-T Therapies	Recruiting
	Nivolumab	Human	NCT03227432	Phase 2 trail in RRMM In Combination of Elotuzumab and Nivolumab with and without Pomalidomide	Withdrawn
			NCT04119336	Phase 2 trail in RRMM In Combination of Ixazomib, Cyclophosphamide, and Dexamethasone	Active, not recruiting
			NCT02903381	A Phase 2 Trial in High Risk Smoldering MM In combination with Lenalidomide and Dexamethasone	Suspended
			NCT02612779	Phase 2 trail in RRMM In combination with Elotuzumab	Completed
			NCT02726581	Phase 2 trail in MM In combination with Pomalidomide and Dexamethasone	Completed
	cemiplimab	Human	NCT03194867	Phase 1/2 trail in RRMM in combination with Isatuximab	Active, not recruiting
PD-L1	atezolizumab	Humanized	NCT03312530	Phase 1/2 trail in RRMM In combination with Venetoclax, With or Without Atezolizumab	Completed
			NCT02431208	Phase 1 trail in MM Alone or in combination with IMiDs and/or Daratumumab	Completed
	durvalumab	Human	NCT03000452 NCT02807454	Phase 2 trail in RRMM In combination with Daratumumab	Completed
			NCT02685826	Phase 1/2 trail in NDMM In combination with Lenalidomide with and without Dexamethasone	Completed
CD47	TTI-622	Human	NCT05139225	Phase 1 trail in MM In combination with Daratumumab Hyaluronidase-fihj	Recruiting
LAG-3	BMS-986016	Human	NCT04150965	Phase 1/2 trial in RRMM.Alone and in combination with Pomalidimide and Dexamethasone	Recruiting
TIGIT	BMS-986207	Human	NCT04150965	Phase 1/2 trial in RRMM.Alone and in combination with Pomalidimide and Dexamethasone	Recruiting
KIRs	IPH2101	Human	NCT00999830	Phase 2 trail in MM of two dose regimens (0.2 and 2 mg/kg)	Completed
			NCT01222286	Phase 2 trail in smoldering MM of two dose regimens (0.2 and 2 mg/kg,every 4 weeks by intravenous route over 1 h, for 6 or up to 12 cycles)	Completed
			NCT01217203	Phase 1 trail in MM In combination of lenalidomide	Completed
			NCT00552396	Phase 1 trail in RRMM	Completed
			NCT01248455	Phase 2 trail in smoldering MM (1 mg/kg) every other month for 6 cycles	Terminated
	lirilumab	Human	NCT02252263	Phase 1 trial in MM.In combination with Elotuzumab	

### Cytotoxic T-lymphocyte antigen-4 (CTLA-4)

CTLA-4 is a 223 amino acid protein belonging to a covalent homodimer of the single V group IgSF with a molecular weight of 24.6 kDa and has CD28 expressed on its surface [20, 21]. Approximately 90% of CTLA-4 is intracellularly located and can exert powerful endocytosis when located in FoxP3 Treg cells or activated primary T cells [22–24].

The mechanism of action of CTLA-4 consists of two main types: one is competitive binding of CD80 (B7-1) and CD86 (B7-2) with CD28 in T cells, which in turn binds to T cell receptor (TCR) signals in antigen-presenting cells, reducing CD28-mediated stimulatory signals and exerting a strong co-inhibitory effect [25]. Another, CTLA-4 can deliver inhibitory signals through the cytoplasmic tail, a process achieved mainly by attenuating nuclear factor of activated T cells (NFAT), activator protein 1 (AP-1) and NF-KB activity, and inhibiting cell cycle protein D3, cell cycle protein-dependent kinase 4 (CDK4) and CDK6 to halt cell cycle progression [26–28]. CTLA-4 also selectively inactivates related kinases and plays an inhibitory role in the IL-2 production pathway, decreasing IL-2 production [26, 29].

As a negative regulator of T cell activation, upregulation of CTLA-4 could also be found in T cells from MM patients and could negatively regulate activated T cells by competitive binding to the costimulatory factor CD80/86 [30]. It has now been confirmed that, CTLA-4 and FOXP3 can be overexpressed in bone marrow samples from patients with newly diagnosed MM [31]. The overexpression of FOXP3 and CTLA4 in BM samples may suggest a suppressed immune response. Two independent studies suggest that genetic variants in the CTLA-4 gene play a role in susceptibility to multiple myeloma and are associated with monoclonal gammopathy of undetermined significance (MGUS), but the exact mechanism needs to be further explored [32, 33].

### Programmed death-1 (PD-1) and programmed death-ligand 1(PD-L1)

PD-1 is a transmembrane protein consisting of 288 amino acids and encoded by the PDCD1 gene on 2q37.3 [34, 35], which can be expressed on the surface of a variety of cells [36–38]. PD-L1 (B7-H1, CD274) and PD-L2 (B7-DC, CD273) are two common ligands of PD-1, both of which can be expressed across the cell surface [39, 40]. PD-L1 can be present in both hematopoietic and non-hematopoietic cells, and its widespread expression can be seen in different cell types. Its expression is regulated by external stimuli [41, 42]. In contrast, PD-L2 expression was less extensive and could only be detected in macrophages, dendritic cells and mast cells [43, 44].

PD-1 expression is not seen in the absence of external stimuli, but can be induced within 24 h after T-cell activation. IFN I and IFN II are now known to effectively drive PD-L1 expression [45], while PD-1 reduces cytokine production, blocks cell cycle arrest, and inhibits the transcriptional process of Bcl-xl [46]. Also, PD-1 inhibits tumor-infiltrating CD4^+^/CD8^+^ T cells, which is an important cause of immune escape of myeloma cells [47].

In healthy individuals, T cells correctly recognize and effectively kill tumor cells. However, myeloma cells exhibit upregulation of PD-L1 protein, which causes apoptosis of T cells when PD-1 on T cells binds to PD-L1, resulting in excessive proliferation of malignant myeloma cells [48–50]. PD-L1 expression levels are higher in MM patients compared with MGUS patients and healthy individuals, and its expression is generally upregulated in relapse or refractory periods. PD-1 is overexpressed on T cells and natural killer (NK) cells of MM patients, and the PD-1/PD-L1 interaction can disrupt the effective anti-myeloma cell immune response and lead to severe immunosuppression and drug resistance [51]. Meanwhile, patients with an increased frequency of PD-1 expression on T cells after autologous stem cell transplantation may have a higher risk of relapse [52]. The preliminary study of our experimental group found that bone marrow mesenchymal stem cells(BMSCs) can inhibit the immune response of CD8 + T cells in multiple myeloma through the PD-1/PD-L1 pathway, and this finding also provides a new idea for the treatment of myeloma [53]. Therefore, blocking the PD-1/PD-L1 pathway in the bone marrow microenvironment with drugs alone or in combination with other therapeutic regimens offers new therapeutic ideas for MM, and has yielded many gains in recent years of intensive research.

### Lymphocyte activation gene-3 (LAG-3)

LAG-3 is a transmembrane protein that was first identified by Triebel's team in 1900 and shown to be inducibly upregulated in activated T cells and NK cells [54]. Its unique KIEELE motif structural domain is closely associated with the regulation of T-cell function [55, 56].

The major ligand of LAG-3 is the class II MHC molecule on antigen- presenting cells (APCs), it cannot be expressed on naive T cells, but can be widely expressed on a variety of cells in response to antigenic stimulation [55], a phenomenon that can be observed on T cells and cytokines with suppressive functions. The ability of LAG-3 expression can be enhanced by IL-2, IL-7 and IL-12 [57, 58], and the increased expression of LAG-3 can be confirmed when the involved regulatory factors, such as nuclear factor of activated T cells (NFAT) [59, 60], show high expression on T cells [61, 62].

Meanwhile, LAG-3, as an NK cell-associated immune checkpoint, targeted inhibition of its expression is beneficial for enhancing NK cell activity, and MM-related clinical treatment studies targeting this feature are currently underway [63]. A currently available prospective study enrolled 71 patients with RRMM, 70 patients with MM, and 70 healthy controls. By measuring CD6 and CD17, as well as cytokines including IL-4, IL-8, TNF-α, and TGF-β, the clinical severity of patients with RRMM was found to be strongly correlated with the frequency of PD-1 and LAG-3 positive T cells, which also implies that LAG-3 and PD-1 are potential biomarkers for the diagnosis of RRMM, directly affecting the prognosis and clinical outcome of patients [64]. A phase I/II randomized trial in 2019 in patients with relapsed refractory multiple myeloma is designed to evaluate two agents, anti-LAG-3 and anti-TIGIT, for their immune effects and safety as single agents and in combination with pomalidomide and dexamethasone. The trial is currently in the recruitment phase and is expected to provide new ideas for the treatment of RRMM.

### T cell immunoglobulin and mucin domain-containing protein-3 (TIM-3)

Tim-3 is a type I transmembrane protein located at 11B1.1 and 5q33.3 of the human genome along with Tim-1 and Tim-4 in the family [65, 66], and has been shown to be expressed on Tregs cells and innate immune cells [67].

Currently known Tim-3 ligands include the following [68, 69]: Galectin-9, Carcinoembryonic antigen cell adhesion molecule 1(Ceacam-1), High mobility histone B1 (HMGB1) and phosphatidylserine (PtdSer).

NK cells exert potent anti-tumor activity, while Tim-3 can negatively regulate NK cell activity. High expression of TIM-3 can occur in the peripheral blood (PB) and bone marrow (BM) of MM patients, but the expression of the three ligands of Tim-3 differs in different myeloma cell lines. Meanwhile, it was confirmed that blocking Tim-3 significantly enhanced NK cell-mediated killing in MM cells in vivo and in vitro [70]. Therefore, the development of NK cell-associated MM immunotherapy regimens based on Tim-3 blockade may have good prospects.

### T cell immunoglobulin and ITIM domain (TIGIT)

TIGIT is an inhibitory receptor expressed on lymphocytes that blocks cell cycle progression at multiple steps and has received attention in recent years as a recent target for tumor immunotherapy [71, 72].

TIGIT has many ligands and is widely present in hematopoietic and non-hematopoietic tissues [73]. As a co-inhibitory receptor that can be expressed on the surface of effector T and NK cells, it binds to the ligands CD155 and CD112 on the surface of myeloma cells (or antigen-presenting cells) by competing with its co-stimulatory counterpart CD226 (DNAM-1) [74]. CD155 (PVR) and CD112 (PVRL2) are highly expressed in a variety of malignancies, including MM [75, 76]. Guilrerey et al. [77] verified that the percentage of CD8 + TIGIT + cells was strongly correlated with myeloma load by in vivo experiments in Vκ*MYC mice, and found that TIGIT was expressed more frequently than other checkpoints by examining CD8 + T cells from human patients, and therefore hypothesized that treatment of wild-type myeloma recipients with an anti-TIGIT antibody could reduce tumor load. A previous study by our group found that TIGIT can appear significantly upregulated on NK cells but downregulated on CD226 in newly diagnosed MM patients (NDMM). The TIGIT ligand, CD155, showed high expression on BMSC but low expression levels on myeloma cells. We confirmed that the CD115/TIGIT signaling pathway plays an important role in the interaction between BMSC and NK cells. Therefore, we speculate that blocking TIGIT could reverse the function of NK cells and provide a new idea for the treatment of MM [78]. A phase 1/2 trial using the TIGIT blocker BMS-986207 in RRMM is currently being recruited to evaluate the efficacy and safety of BMS-986207 alone and in combination with Pomalidimide and Dexamethasone.

### V-domain immunoglobulin suppressor of T cell activation (VISTA)

VISTA characterized by a family of B7 and CD28 immunomodulatory molecules, is a type I transmembrane protein that acts as both a ligand and a receptor [79–81]. In humans, VISTA is predominantly expressed in hematopoietic tissues [82] and has now been shown to be highly expressed in myeloma cells. There are two receptors for human VISTA, PSGL-1 and VSIG3, which upon binding can exert immunosuppressive functions. In addition to these, there is a receptor VSIG8, but its exact mechanism of action has not been fully determined [83, 84]. One study comparing PB and BM of MM patients with healthy individuals found a significant increase in the percentage of VISTA co-expressed with PD-1, Tim-3 and TIGIT in CD3 + , CD4 + , CD8 + and Treg cells in MM patients. The high expression of VISTA, PD-1, and Tim-3 can be evident on T cells In MM patients, especially in PB, suggesting T cell exhaustion and dysfunction. Therefore targeting VISTA has the potential to reverse T-cell depletion in MM and improve T-cell function [85]. A 2021 study [86] analyzed transcriptomic data from a cohort of 718 patients from independent trials and 1654 bone marrow samples from eight clinical trials and concluded that combined VISTA + , CD11b + , and CD8 + cell scores can be used to assess the prognosis of MM and to guide immunotherapy stratification of MM patients.

### B and T lymphocyte attenuator (BTLA)

BTLA is localized in the q13.2 region of chromosome 3 [87] and belongs to the CD28 superfamily. BTLA is highly expressed in lymphoid organs, mainly in T cells and B cells [88, 89].

Previous studies have found that the herpesvirus entry mediator (HVEM) is its only ligand in human cells [90]. The mechanism of action of BTLA is mainly related to the PI3K signaling pathway, which transduces inhibitory signals by recruiting SHP-1 and SHP-2 to T cells, causing downregulation of TCR signaling [91]. In addition, upon binding to HVEM, it activates the NF-κB pathway and conducts pro-inflammatory and pro-survival signals. Similar to other inhibitory receptors including PD-1, Tim-3, and LAG-3, BTLA exhibited high expression in MM and suggested T-cell exhaustion and dysfunction [92, 93]. A 2015 randomized trial (NCT01319422) [94] evaluated the efficacy of continuous or intermittent administration of pomalidomide/dexamethasone in the treatment of patients with lenalidomide-resistant myeloma. These results suggest that pomalidomide leads to an increased ratio of Tim-3 ( +) NK cells and BTLA ( +) T cells, exerting a co-inhibitory effect and inducing T cell activation.

### Killer immunoglobulin-like receptors (KIRs)

KIRs are a family of cellular receptors. Members of the activating family are called KIRxDS with a short cytoplasmic ITAM activation signaling domain, while members of the inhibitory family are called KIRxDL with a long signaling domain [95].

KIRs are predominantly expressed on NK cells and can occasionally be low expressed on T cells. The receptors for KIRs on NK cells are diverse in expression, with the four most common inhibitory receptors being KIR2DL1, KIR2DL3, KIR3DL1, and KIR3DL2 [96]. Currently available data indicate that NK cell licensing is disrupted in myeloma, resulting in a weakened affinity for KIR-ligand interactions. When the expression of inhibitory NK ligands on myeloma cell targets increases, NK cells will gradually lose their ability to recognize tumor cells, leading to a loss of immune surveillance [97, 98]. It has been shown that KIR2DL1 expression is higher in patients with myeloma than in healthy individuals; KIR2DS4 and KIR2DS5 suggest a high prevalence of MM. In addition, high expression of KIR3DS1 is closely associated with shorter progression-free survival in patients with MM [99, 100].

In addition to the several common immune checkpoint inhibitors introduced above, there are many newly discovered immune checkpoint inhibitors, such as KIR, NKG2A, IDO1, TDO2, and 4-1BB. Their mechanism in MM is unclear, and further research is needed to better serve clinical practice.

## Treatment modalities for immune checkpoint

### Covering immune checkpoint with monoclonal antibodies

**Fig. 3:**
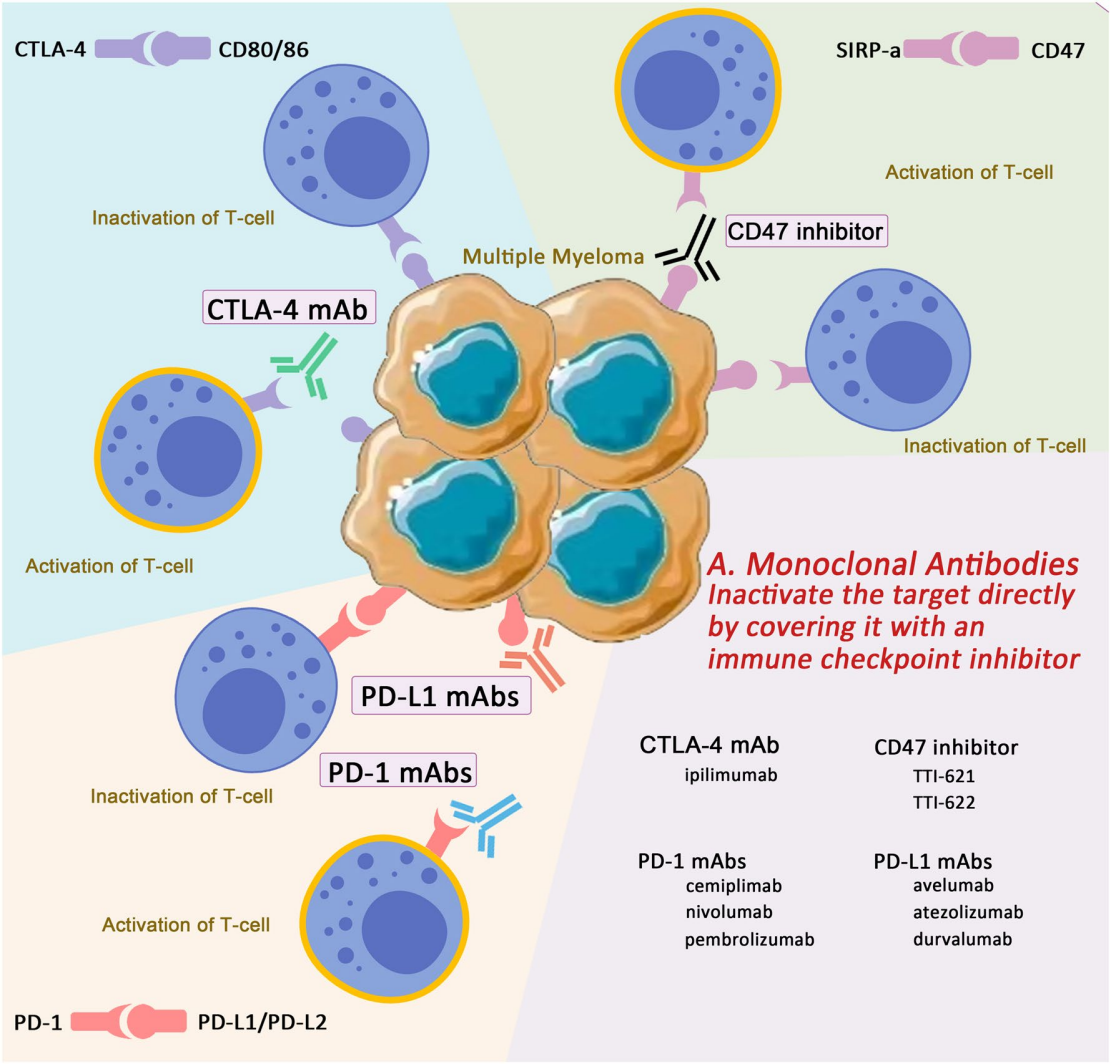
Covering immune checkpoint with monoclonal antibodies. ICIs aim to block co-inhibitory pathway. The main targeted immune pathways include the PD-1/PD-L1, CTLA-4/B7, and CD47/SIRP-a signal pathways. From 2011 to date, there have been seven FDA-approved and marketed ICIs that can be used in clinical treatment, including one CTLA-4 mAb (ipilimumab), three PD-1 mAbs (nivolumab, pembrolizumab, and cemiplimab), and three PD-L1 mAbs (atezolizumab, avelumab, and durvalumab)

Immune checkpoints are important pathways in the immune system that exert inhibitory effects and are dominated by receptor/ligand mechanisms. The presence of this structure is extremely important for maintaining autoimmune tolerance and regulating physiological immune responses, and for preventing the immune system from damaging and destroying normal tissues and organs as a result of excessive immune responses. However, tumour cells undergo immune escape through the 'cancer immune cycle', and thus the therapeutic application of monoclonal antibodies targeting immune checkpoints came into being at this moment in history, providing a major breakthrough in cancer treatment [12, 13]. ICIs aim to block co-inhibitory pathways, release pre-existing anti-tumour immune effectors, reset or restore dysfunctional effector T cells, and thereby promote immune-mediated tumour cell clearance. The main targeted immune pathways include PD-1/PD-L1, CTLA-4/B7 and CD47/SIRP-a signalling pathways [101]. In contrast to conventional anti-tumour therapies, ICIs enhance the anti-tumour effects of the host immune system and maintain a balance between anti- and pro-inflammatory signals [102]. Since 2011, seven FDA-approved and marketed ICIs have been made available for clinical use, including one CTLA-4 mAb (ipilimumab), three PD-1 mAbs (nivolumab, pembrolizumab, and cemiplimab), and three PD-L1 mAbs (atezolizumab, avevelumab, and durvalumab) [103]. Of these, ICIs have been used in multiple clinical trials or have been approved for use in patients with MM (Fig. 3).

#### CTLA-4 inhibitor

Both CTLA-4 and CD28 bind to CD80 and CD86 ligands, whereas competitive binding of CTLA-4 to the ligands inhibits T-cell activation and prevents CTLA-4 from exerting its anti-tumour effects. Ipilimumab, a CTLA-4 inhibitor, was approved by the FDA in 2011 as the first ICI and has been successfully used in the treatment of melanoma and has been shown to be effective against a variety of haematological malignancies [104]. However, enrolment is ongoing in a trial (NCT02681302) of concurrent use of two checkpoint inhibitors (ipilimumab and nivolumab) in patients with MM and lymphoma [105].

#### PD-1/PD-L1 inhibitor

Correlative testing of plasma from myeloma patients has shown that high expression of PD-L1 is associated with high expression of PD-1 receptors on T cells and NK cells. When PD-L1 on myeloma cells binds to PD-1 receptors on immune cells, it prevents the production of Th-1 cytokines, leading to immune dysfunction [106, 107]. Numerous experiments have been conducted for the PD-1/PD-L1 axis, and drugs have been developed to target PD-1 and PD-L1, respectively [108]. Pembrolizumab monotherapy failed to show a significant effect in RRMM and only supported patients to achieve disease stabilisation. In RRMM (n = 27), the best response to navul monotherapy was disease stabilisation (63%), and one patient fully recovered from a single bone lesion with radiotherapy (4%). These studies emphasise that monotherapies targeting the PD-1/PD-L1 axis alone have limited efficacy and that these mAbs may need to be used in combination with other agents to achieve clinical efficacy.

#### CD47 inhibitor

In addition to the two common pathways mentioned above, ICIs targeting the CD47/SIRP-a pathway have also entered clinical trials. CD47, a pentameric transmembrane protein normally expressed on tumour cells, binds to SIRP-a, a signal-regulating protein found on macrophages, to produce a “don’t eat me” signal that ensures that the tumour cell escapes the macrophage's immune surveillance. On the one hand, the macrophage is unable to directly phagocytose the tumour cell; on the other hand, it is unable to deliver the “alien” signal to the T cell. Therefore, the scientists found that the CD47 antibody can block its binding to SIRP-a, thus activating the killing effect of macrophages on tumour cells [109, 110]. TTI-621 and TTI-622 are two new drugs currently being developed to target CD47 and are currently in Phase Ia/Ib in clinical trials [111, 112]. The phagocytic effect of TTI-621 on various haematological and solid tumour cells has been used to inhibit the growth of B-cell lymphoma xenografts and acute myeloid leukaemia (AML) in preclinical studies (NCT02890368, NCT02663518). However, this has been accompanied by adverse drug reactions, the most common of which may occur in different systems [113]. Recently, the FDA approved a clinical efficacy study (NCT05139225) testing the combination of TTI-622, daratumumab, and hyaluronidase-fihj in patients with RRMM and a fully human anti-CD47 monoclonal antibody study (NCT03512340). Meanwhile, a Phase I study (NCT03512340) in patients with solid cancers and haematological tumours is ongoing [112].

### Regulating the expression of immune checkpoints

**Fig. 4:**
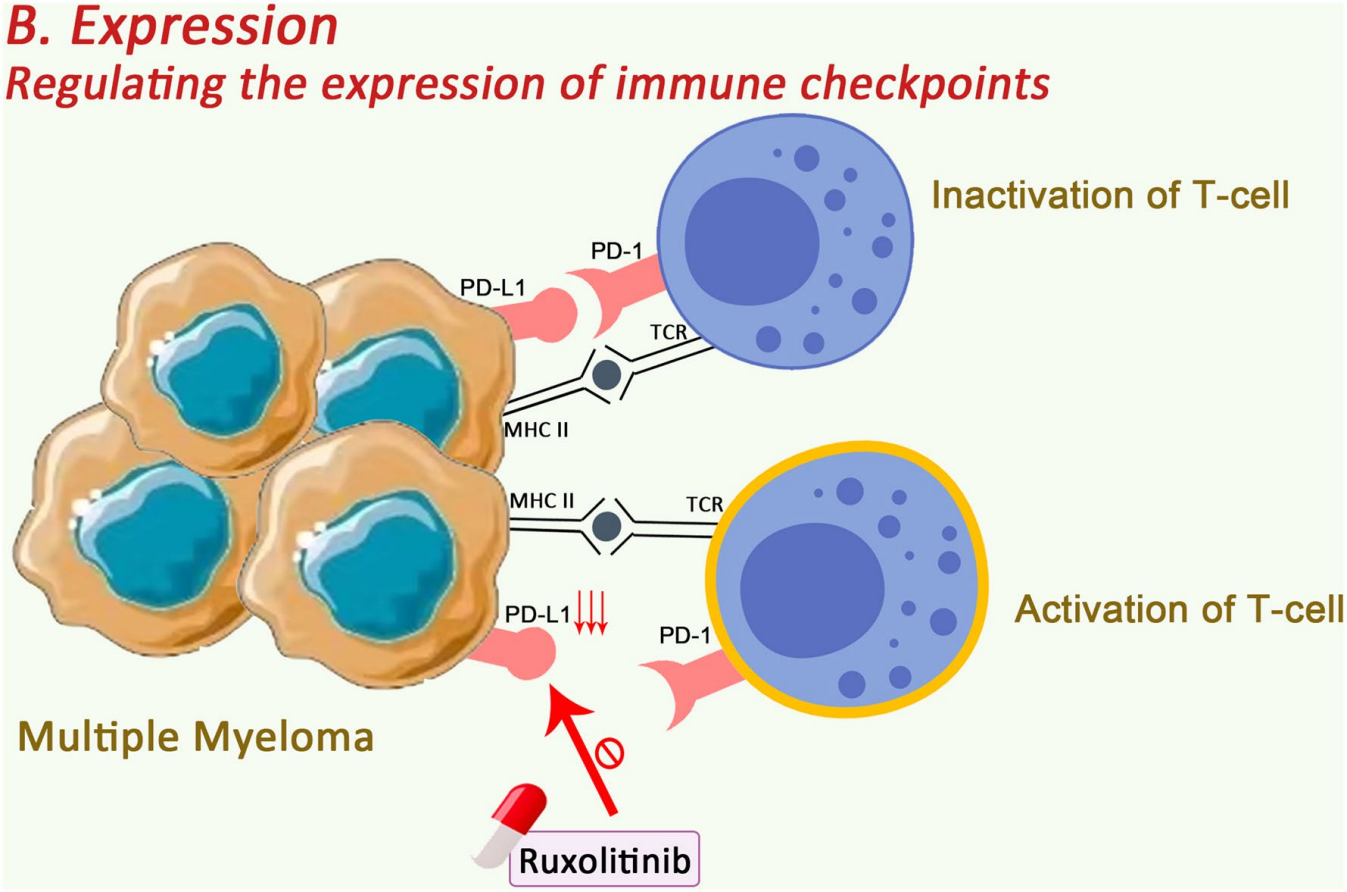
Ruxolitinib can block PD-L1 expression, thus enhancing the anti-MM effect of T cells.So Altering the expression of immune checkpoints may provide a new idea for immunotherapy of MM

Immune checkpoints create conditions for tumour cells to evade surveillance by the immune system, which can be seen in MM with abnormal immune checkpoint expression. Therefore, altering immune checkpoint expression offers ideas for immunotherapy in MM. Ruxolitinib (RUX), an inhibitor of the Janus kinase (JAK) family of protein tyrosine kinases, is approved for the treatment of myeloproliferative disorders. In 2021, Chen and his team [114] found that Ruxolitinib could block the expression of PD-L1 in in vitro experiments, thus enhancing the anti-MM effect of T cells. Down-regulation of PD-L1 expression in solid tumours is also clinically important for enhancing immune responses. Several studies on solid tumours have found that altering the expression of immune checkpoints enhances anti-tumour immunity; patients with KRAS-mutant lung cancer have shown significant efficacy with luteolin and its derivative apigenin, and the enhancement of their anti-tumour ability has been associated with the down-regulation of PD-L1 expression [115]; Cilibin overcomes PD-L1-mediated drug resistance in nasopharyngeal carcinoma by down-regulating PD-L1 expression [116]; KYA1797K down-regulates PD-L1 and blocks immune escape in colon cancer stem cells by inhibiting the β-catenin/STT3 signalling pathway [117]; elraglusib reduced the expression of PD-1, LAG-3 and TIGIT and enhanced the cytolytic killing of melanoma cells by CD8T cells [118]. However, the regulation of the expression level of other immune checkpoints is not clear; nevertheless, this approach may be a new way to enhance anti-tumor immunity (Fig. 4).

### ICIs in combination with other modalities in multiple target therapy

Current treatments for MM include proteasome inhibitors, immunomodulators, specific antibodies, and cellular therapies. Unfortunately, for many patients, treatment is limited and ends in relapse or ineffectiveness. The median event-free survival (EFS) and OS for patients with treatment-refractory MM with IMiDs and bortezomib are only 5 months and 9 months, respectively [119]. Combination therapy with multi-targeted inhibitors appears to produce better efficacy and synergistically promote control of myeloma tumour cells compared to monotherapy. The combination of myeloma-targeted and immune-targeted drugs offers a new diagnostic and therapeutic approach.

#### ICIs + immunomodulators (IMiDs)

**Fig. 5:**
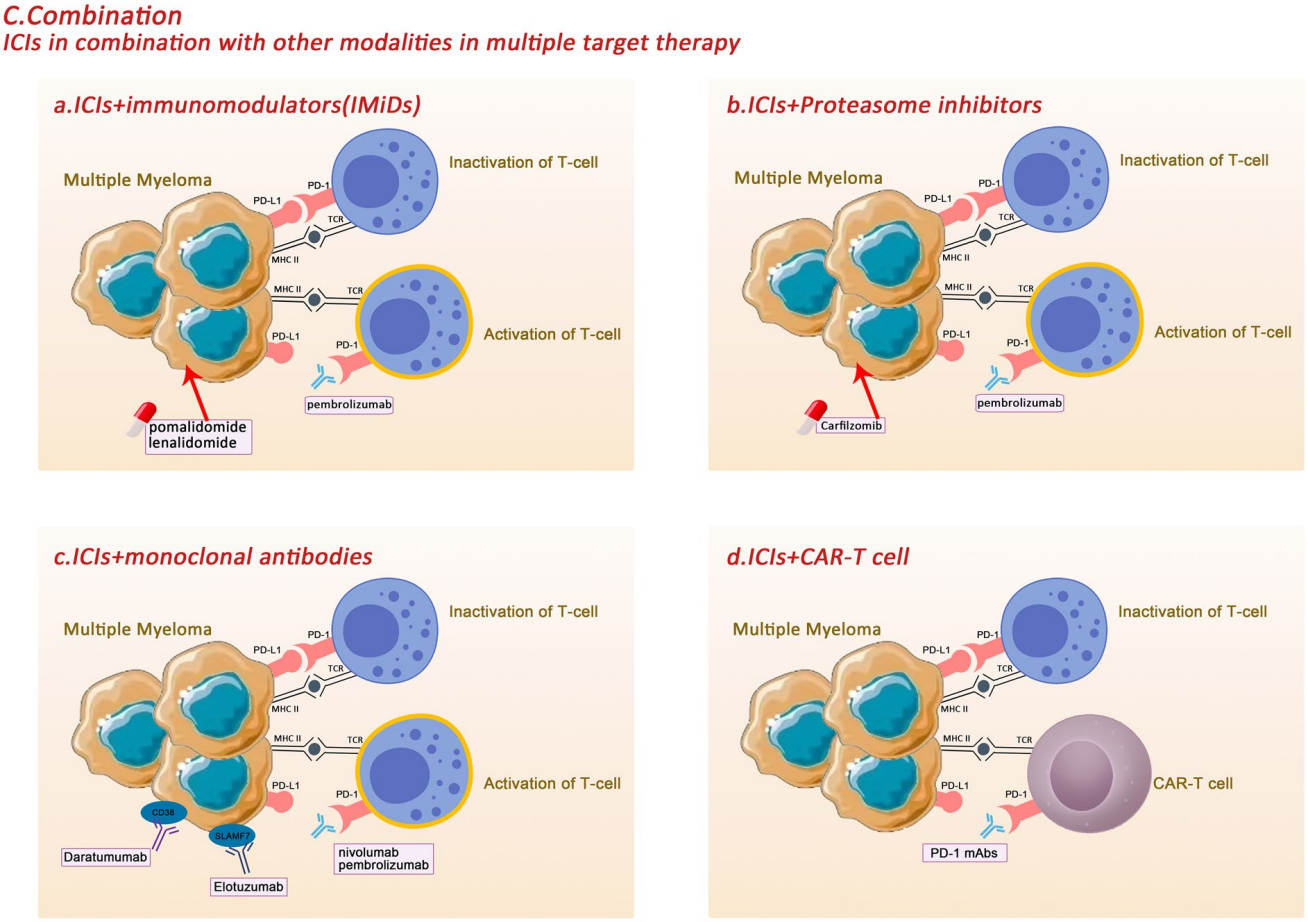
**A** The combination of bonadomide, lenalidomide and ICIs showed great therapeutic efficacy in myeloma cells. **B** Carfilzomib enhanced the efficacy of PD-1 in egulating the bone marrow microenvironment and played a role in eliminating the tumor. **C** The combination of MM-specific antibodies and ICIs may have potential role in the treatment efficacy for MM. **D** Studies have combined CAR-T therapy with PD-1 blockade and have shown good therapeutic efficacy

IMiDs-mediated regulation of haematopoietic cell transcription factors and repressors involving Aiolos and Ikaros plays an important role in cellular physiological processes. By down-regulating interferon regulatory factor 4 (IRF4) and c-Myc, the production of IL-2, IL-10 and IFN-γ in T cells was reduced on the one hand, while on the other hand, the activation of T cells and NK cells was promoted, the production of Tregs in vitro was inhibited, and apoptosis of myeloma cells was induced [120, 121]. This result was validated in a preclinical model in transgenic mice by producing thalidomide metabolised derivatives to degrade Ikaros and Aiolos [122]. Therefore, several studies have tested the combination of peblizumab and IMiDs to determine whether triple therapy improves clinical efficacy. One study (trial NCT02289222) confirmed the efficacy of bonadomide and lenalidomide in enhancing ICIs in myeloma cells, while the combination of dexamethasone, lenalidomide, and the PD-L1 antibody pembrolizumab was efficacious in patients with lenalidomide-refractory myeloma, with an objective response rate (ORR) of 76% [123]. However, the combination also enhances drug toxicity. Preliminary data suggest a higher incidence of toxicity in the pembrolizumab treatment group, particularly immune-mediated toxicity (including hyper/hypothyroidism, colitis and skin reactions) [124]. A phase I multicentre, multi-cohort study (NCT02036502) combining the PD-1 inhibitor pembrolizumab, lenalidomide, and low-dose dexamethasone in patients with RRMM found that 62 patients had an ORR of 44% (defined as sCR + very good partial remission (VGPR) + partial remission (PR)). However, 59.7% of these patients experienced grade 3 or higher adverse events and 3.2% died. Subsequently, an interim analysis by an external Data Monitoring Committee (DMC) of two Phase III randomised trials of pomalidomide and low-dose dexamethasone in combination with or without Pembrolizumab (NCT02576977, NCT02579863) found that the condition of these patients may have worsened following combination therapy with Pembrolizumab. The risks of combining Pembrolizumab outweigh the potential benefits for patients with MM [123]. There have also been several clinical trials of anti-PD-L1 antibodies as monotherapy or combination therapy in patients with plasma cell myeloma, but most of these trials have been discontinued for a variety of reasons.The combination of nivolumab with drugs such as lenalidomide did not show significant effectiveness [125]. The immunotoxicity of the combination is a problem for such treatments that needs to be addressed (Fig. 5a).

#### ICIs + Proteasome inhibitors

Carfilzomib is a proteasome inhibitor that disrupts cellular protein homeostasis by irreversibly inhibiting the proteasome at the ChT-L site. Because of its ability to secrete and produce large amounts of proteins, carfilzomib has been shown to have significant efficacy in patients with MM and was approved by the U.S. Food and Drug Administration for use in RRMM in 2012 [126]. Subsequent studies confirmed that proteasome inhibitors partially induced the unfolded protein response (UPR), which encodes the expression of M1-associated cytokines by M2 macrophages, and increased the expression of CD68, MHC II, and the costimulatory molecules CD80 and CD86 [127]. In vivo experimental studies in transgenic mice conducted by Zhou et al*.* [128] found that regulating the bone marrow microenvironment with carfilzomib enhanced the efficacy of PD-1 and played a role in eliminating the tumor in lung cancer. A study attempting to combine pembrolizumab with low-dose dexamethasone and carfilzomib in patients with RRMM has progressed in cohort 2 of the phase I study (KEYNOTE-023). Ten patients with RRMM were enrolled in the trial and given a median number of cycles of drug therapy each. The results found that six of these patients developed grade 1 AEs, with an ORR of 70% and a median PFS and OS of only 14.3 and 22.5 months, respectively [129]. Although the study was not successful, the treatment modality of PD-1 inhibitors combined with proteasome inhibitors may remain a potential therapeutic option for patients with MM (Fig. 5b).

#### ICIs + monoclonal antibodies

Several monoclonal antibodies specific to MM have been developed, including (1)BCMA: Belantamab, (2)GPRC5D: Talquetamab, (3)CD38: daratumumab, isatuximab, MOR202, (4) SLAMF7: elotuzumab, (5) CD56: lorvotuzumab, (6) CD138: indetuximab [130, 131].

SLAMF7 is expressed on myeloma cells and NK cells. Its corresponding monoclonal antibody, elotuzumab, cuts off myeloma cells from bone marrow stromal cells, enhances the action of NK cells, mediates antibody-dependent cell-mediated cytotoxicity (ADCC) and exerts anti-tumour effects. Studies have been conducted combining elotuzumab with other drugs.A phase I study [132] showed that combining elotuzumab with dexamethasone and lenalidomide in RRMM resulted in a higher remission rate (at least partial remission, 82%), which was significantly higher compared with that achieved by the lenalidomide and dexamethasone combination (remission rate of 60%). In another randomized phase III study (ELOQUENT-2) [133], the remission rate in patients treated with the three drugs was higher than that in patients treated with lenalidomide and dexamethasone (79% vs. 66%). Furthermore, in a phase I study [134], the use of the triple combination of elotuzumab, bortezomib, and dexamethasone in RRMM showed an ORR of 48%, with a median time to progression of 9.5 months; correspondingly, in the phase II study [135] with the triple combination, the median PFS in the experimental group was longer than that in the control group (9.7 months vs. 6.9 months), and the overall response rate was also higher (66% vs. 63%). In a previous study, we found that the drug combination with elotuzumab showed good clinical efficacy. A preclinical study in mice confirmed the clinical benefits of elotuzumab in combination with anti-PD-1. Phase III clinical trials (NCT02726581) [136] are currently ongoing in MM. In this trial, patients using only pomalidomide and dexamethasone were used as the control group, patients using a combination of nivolumab, pomalidomide and dexamethasone as experimental group 1, and patients using a combination of nivolumab, elotuzumab, pomalidomide and dexamethasone as experimental group 2, and the safety and efficacy of the combined treatment regimens were evaluated separately. The trial was successfully completed in April 2022, and we hope that the analysis of the results associated with this study will lead to new innovations in treatment options and new treatment concepts for MM patients.

CD38, a transmembrane glycoprotein highly expressed in malignant plasma cells, is the first anti-CD38 monoclonal antibody for immunotherapy. A 3-year pooled analysis showed that monotherapy could treat RRMM patients with a median OS of 20.5 months and an ORR of 30.4% [137, 138]. In a I/II, phase I study [139] confirmed that combining lenalidomide, daratumumab, and dexamethasone improved the ORR of 81% in RRMM. Another randomized III, phase I, study (POLLUX) [140] comparing the triple combination with lenalidomide and dexamethasone only in RRMM showed significantly enhanced efficacy. These two classes of monoclonal antibodies act primarily through T- and NK-cell-mediated cytotoxic effects, and may be able to significantly improve efficacy by releasing immune effector cells from the inhibitory effects of the PD-1/PD-L1 axis. Experimental results from preclinical solid tumour models suggest that the combination of anti-PD-1 and daratumumab may have an interactive effect. Clinical trials of daratumumab in combination with nivolumab/pembrolizumab have also been conducted in patients with RRMM (NCT02431208, NCT01592370 and NCT03357952) [141]. Based on trials in solid tumours, we anticipate that the combination of such drugs in MM patients will produce correspondingly excellent efficacy.

Further explorations in the research phase include [142]: phase 1 trial of vibostolimab (TIGIT mAb) combined with pembrolizumab in metastatic NSCLC; phase 1/2 trial of anti-CD137 mAb with nulliumab in solid cancer and B cell non-Hodgkin’s lymphoma; and a phase 1 trial of nulliumab with second-generation anti-KIR mAb ibrutinib in patients with lymphoma and myeloma;and an experiment combining navulizumab and ipilimumab for melanoma [143]. While the combination further improved OS and PFS, it also increased the incidence of immune-related toxicity compared with that for monotherapy with each agent. Although many experiments are still in the exploratory stage, the combination of MM-specific antibodies and ICIs have potential role in the treatment efficacy for MM (Fig. 5c).

#### ICIs + cell therapy

Chimeric antigen receptor-T (CAR-T) cell therapies have now shown promising clinical efficacy and are approved for the treatment of haematological tumours. A CAR consists of three extracellular antigen-binding domains of variable antibody single-chain fragments, a transmembrane domain and an intracellular signalling or costimulatory domain (usually CD3ξ). The latest fourth-generation CARs contain CD28, 4-1BB and OX-40 stimulatory structural domains and express cytokines [144, 145]. However, the serious adverse effects associated with CAR-T therapies when used alone present new challenges for patients and their clinical risks have not been addressed. CAR-T therapies have been studied in combination with PD-1 blocking therapies and have shown promising efficacy in mouse models of lung cancer xenografts, hepatocellular carcinoma, metastatic melanoma, and follicular cell lymphoma. Experimental combinations of CAR-T and anti-PD-1 have progressed primarily in lymphoma (NCT04134325, NCT04213469, NCT03932955, NCT03540303, NCT04163302, NCT03298828, NCT03287817, NCT03208556, and NCT02650999).NCT04162119 is a trial starting in 2019 to treat patients with RRMM with BCMA- PD1-CART treatment and evaluate its efficacy [146] (Fig. 5d).

For patients with haematological tumours, autologous haematopoietic stem cell transplantation (ASCT) is an important measure to achieve radical cure of the disease; however, there are very limited therapeutic options available for patients who are unable to undergo transplantation or who have relapsed after transplantation. The current Phase I study (KEYNOTE-013) of pembrolizumab in patients with relapsed/refractory cHHL (RRcHL) demonstrated its safety and feasibility in RRcHL, which was confirmed by a subsequent Phase II study (KEYNOTE-087). A Phase II trial of pembrolizumab for post-ASCT consolidation in RRcHL patients (NCT02362997) is in the clinical study phase. In addition, it is hypothesised that this treatment may result in favourable PFS for high-risk RRcHL patients [147]. Similarly, experimental studies have been conducted in the field of myeloma. In 2018, researchers presented a study of the efficacy of post-transplant anti-PD-1 therapy, which showed that a maintenance regimen of pembrolizumab in combination with lenalidomide is feasible in the early post-ASCT period, but the efficacy of the regimen is uncertain and further studies are still needed to obtain data to support it [148]. In addition, NCT02906332 is an open-label phase II single-centre trial of pembrolizumab, lenalidomide and dexamethasone in patients with high-risk multiple myeloma (hrMM) following high-dose chemotherapy combined with autologous stem cell transplantation (ASCT). The trial explores the relevance of immunological analysis by collecting bone marrow fluid and peripheral blood samples from patients before and after treatment, in the hope of providing new treatment options for this group of hrMM patients.

In conclusion, combining ICIs with other therapies, including immunomodulators, proteasome inhibitors, and specific mAbs, results in favourable outcomes, and a large number of relevant studies are currently underway that will provide further evidence and new benefits for patients with MM.

### Selection of both “broad-spectrum” and “narrow-spectrum” targeted therapies

In the early stages of treatment where no specific immunopathogenesis is defined, “broad-spectrum” therapies may be used to provide an initial integrated or generalised approach targeting processes such as the adenosine axis, inflammatory cytokine storms, and tumour metabolites that can lead to immune disturbances in MM. Better efficacy may be observed when combined with immune checkpoint inhibitors.

1. Adenosine belongs to a group of immunosuppressive metabolites that are widely present in the tumour microenvironment and play a corresponding role in the pathogenesis of MM. Therefore, several studies have considered the combination of adenosineergic axes (including CD39 mAb, CD73 mAb, A2aR mAb) and ICIs (PD-1/PD-L1 mAb, CTLA-4 mAb, TIGIT mAb) for the treatment of tumours [149]. In preclinical studies in solid tumours, ICIs such as CTLA-4, PD-1/PD-L1 and IDO-1 were blocked from binding to the adenosine axis and were found to be effective in restoring T-cell function and producing anti-tumour effects [150].
2. Inflammatory factor storm is an overreaction of the immune system to an external stimulus, resulting in excessive mutation of the immune system. In MM, cytokines and chemokines, immune cells, and other markers may be abnormal and can be predictive of a patient's prognosis [151]. Overproduction of cytokines in MM stimulates RANKL-mediated osteogenesis while inhibiting osteoblastic differentiation of bone marrow stromal cells, leading to extensive bone destruction and rapid bone loss, which greatly affects patient survival time [152]. In MM, the Th1/Th2 ratio is severely imbalanced, Tregs are unable to control T cell proliferation and function, and Th17 cells increase in response to increased pro-inflammatory cytokines [153, 154]. Cytokines, including IL-2, IL-17, etc., play an important role in myeloma cell proliferation, metastasis and drug resistance [155, 156]. Therefore, patients can benefit from immune-related therapies (e.g. immunomodulators) to improve the immune microenvironment in MM.
3. Tumour metabolites can act as bioregulators to promote tumour cell growth and proliferation through cellular metabolism recoded by tumour cells. Intrinsic metabolic pathways play important roles in supporting bioenergetics, biosynthesis, and promoting epigenetics and protein expression. Similar to most solid tumours, glucose and glutamine metabolism have been most studied in MM, and relevant targeted therapeutic strategies have been developed for these two metabolic pathways. At the same time, it has been found that MM resistance to proteasome inhibitors is associated with the upregulation of metabolic pathways in which mitochondria also play an important role, and therefore targeted therapies for these pathways are expected to break the barrier of MM resistance to proteasome inhibitors [157, 158].

**Fig. 6:**
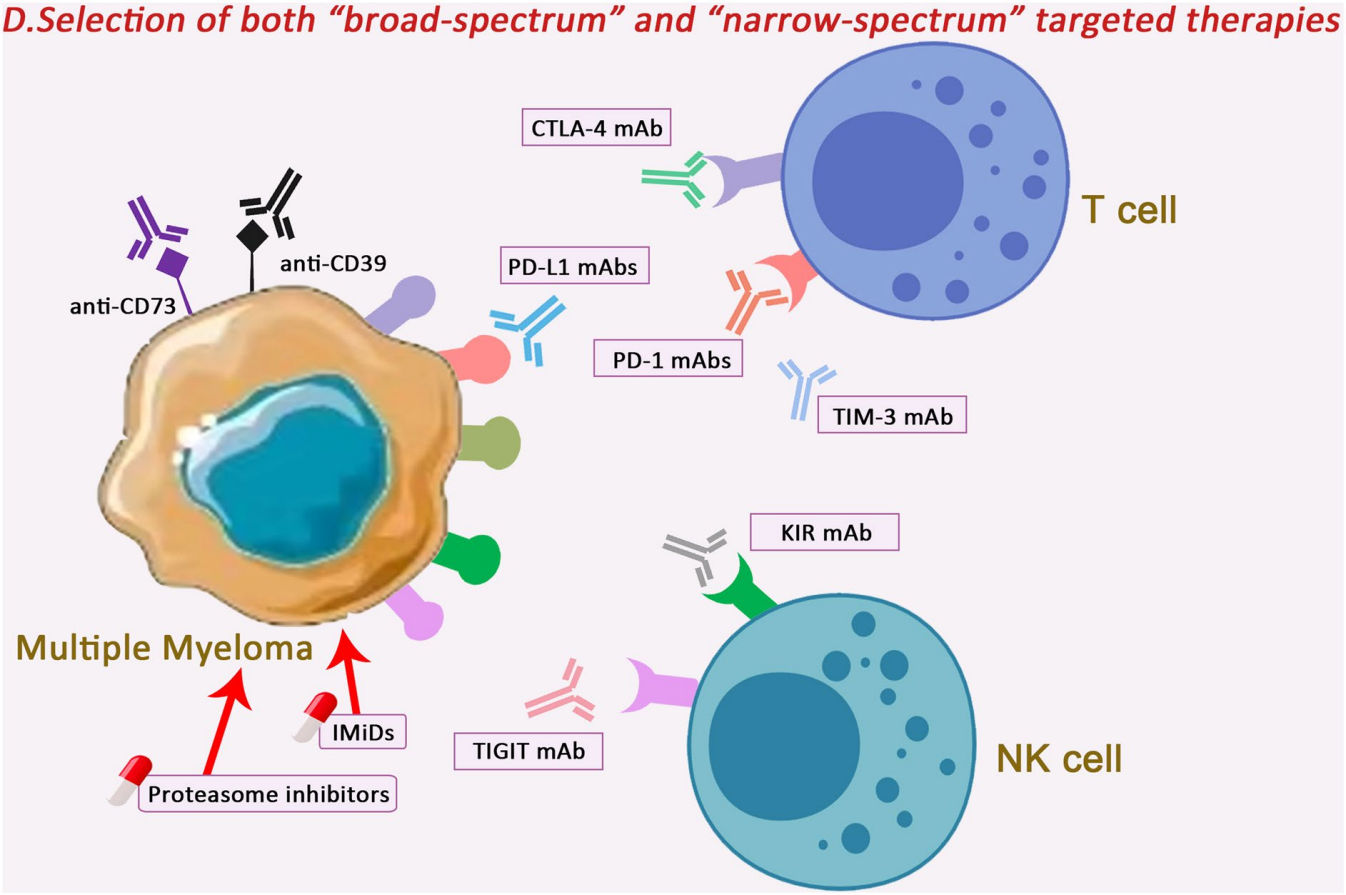
“Broad-spectrum” treatment can be used in the beginning of treatment without clear specific immune pathogenesis to provide an initial comprehensive or generalized approach for processes that can cause MM immune disorders, such as adenosine axis, inflammatory cytokine storm, and tumor metabolites.While “narrow-spectrum” therapy can target to relevant cells after clarifying the mechanisms of immune abnormalities

Unlike “broad-spectrum” therapies, “narrow-spectrum” therapies can be targeted to the relevant cells after the mechanism of immune abnormality has been clarified. Accordingly, patients can be treated with targeted therapy or a combination of multiple therapeutic regimens according to the different expression levels of immune checkpoints on various cells, thereby maximising the clinical efficacy of the drugs and enabling patients to achieve greater clinical benefits. When patients have multiple immune checkpoint abnormalities at the same time, due to the clinical difficulty of treating all abnormal checkpoints at the same time, targeted therapy for the checkpoints with the most pronounced abnormal expression will generally be selected and combined with other treatment options, such as immunomodulators, in order to improve the efficacy. For MM patients who present with different cellular subpopulations in a state of suppression and depletion, specific treatments may be directed at different immune checkpoints on different cells. As mentioned above, for patients with NK cell abnormalities, TIGIT, KIR and NKG2A on their surface can be targeted, while for T cells, CTLA-L4, PD-1 and TIM-3 checkpoints on their surface are mainly targeted. A large number of preclinical and clinical studies have explored the therapeutic options of ICIs in combination with other therapeutic approaches in solid tumours and haematological neoplasms, but the data from relevant studies in MM are still scarce, and further studies are needed to determine their therapeutic options (Fig. 6).

### Precision treatment

MM cells have aberrant expression of multiple immune checkpoints, with varying levels of expression in different cell subpopulations. Therefore, the concept of precision therapy was born. For MM patients with different expression, we can target the corresponding ICIs for treatment, thus improving clinical outcomes [159]. The concept of precision therapy can accordingly be summarised in two ways. On the one hand, it is multi-targeted therapy against abnormally expressed immune checkpoints; on the other hand, it can also be formulated as targeted therapy against subpopulations of abnormal immune cells caused by immune checkpoints.

Most MM patients exhibit multiple immune checkpoint abnormalities rather than a single immune checkpoint abnormality, and in clinical treatment, only one of the most clearly expressed immune checkpoints can be blindly targeted, and therefore the therapeutic effect is often not particularly significant. However, there are not many case reports of precise combination therapy directly targeting multiple abnormally expressed immune checkpoints in clinical treatment. Currently, more MM-related combinations have focused on the combination of IMiDs and ICIs, and it has been found that IMiDs therapy decreases PD-1 expression on NK and T cells, as well as PD-L1 expression on myeloma cells, and that the down-regulation of PD-1/PD-L1 expression leads to a compensatory up-regulation of other immune checkpoints [160]. This makes combination therapy difficult. Other modalities of combination therapy have been undertaken in the field of MM, as described above, and are expected to progress accordingly. In the field of solid tumours, studies have been conducted on combination therapy with immune checkpoints abnormally expressed on NK cells. However, there are no research results on the treatment strategy of MM, and we expect that more attention will be paid to this area of research and corresponding research results will be obtained, which will provide new ideas for clinical treatment.

**Fig. 7:**
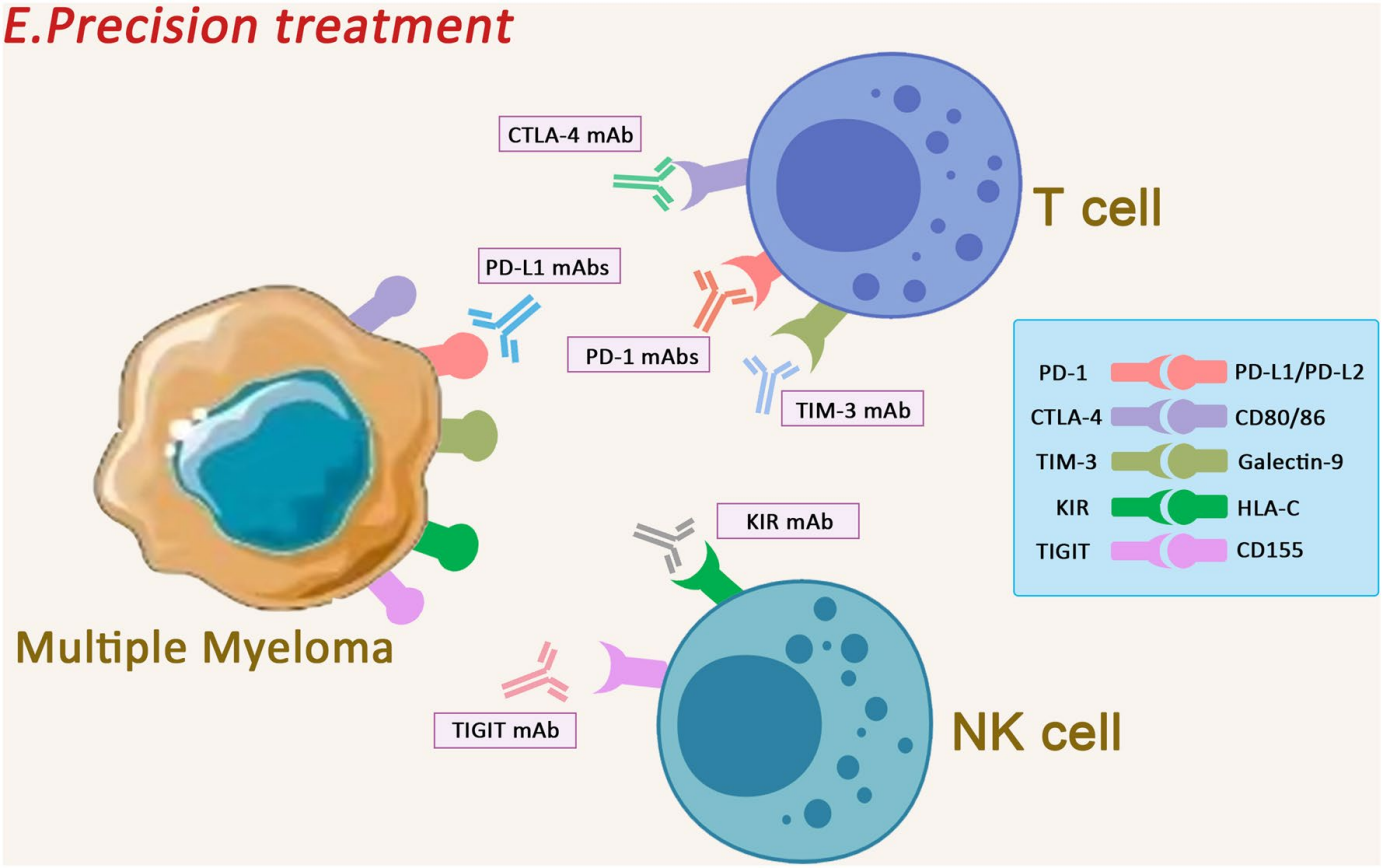
Precision treatment. Existing research results targeting NK cell immune checkpoint therapy mainly include preclinical studies of anti-CD96 and anti-TIGIT and clinical studies of anti-KIR and anti-NKG—2A. And the antibodies targeting T-cell immune checkpoint inhibitors, predominantly related to CTLA-4, PD-1 and TIM-3

MM patients also frequently have abnormal expression of different immune checkpoints on different immune cell subsets such as NK cells and T cells. Existing studies targeting NK cell immune checkpoint therapy have focused on preclinical studies focusing on anti-CD96 and anti-TIGIT and clinical studies focusing on anti-KIR and anti-NKG-2A [161]. Meanwhile, antibodies targeting T-cell immune checkpoint inhibitors, seem to be predominantly related to CTLA-4, PD-1 and TIM-3 [162]. Below we provide a specific overview of the precise therapeutic options related to the relevant immune checkpoints on NK and T cells (Fig. 7).

The main drugs developed to target the KIR2D-L1/2/3 NK inhibitory receptors are IPH2101 and lirilumab (IPH2102 /BMS-986015), which have been used in clinical trials. In a phase I experiment (NCT00552396) [163], it was found that IPH2101 reduced NK cell responsiveness in MM patients and that monotherapy was more effective. When combined with lenalidomide (NCT01217203), lenalidomide enhanced NK cell function and upregulated NK cell surface receptors in MM, which deserves the evaluation in a clinical study [164]. A phase I study of lirilumab failed to find any definite clinical efficacy in patients with MM [165], and many studies on the combination of KIR blockade with anti-CD20 antibody (NCT02481297), and with anti-PD-1 antibody and 5-azocytidine (NCT02599649) are ongoing, including phase I experiments (NCT2252263) with elotuzumab and lirilumab for the treatment of MM.

Immune surveillance and treatment in MM have been shown to be associated with CD226 released by NK and T cells, accompanied by the upregulation of CD155, where TIGIT expression can be increased in CTL and NK cells. CD8 T cells highly express TIGIT on the surface, often suggesting MM progression, which has been verified in mouse models of solid tumors (e.g., colorectal) [166]. In addition, some in vitro experiments found that low expression of TIGIT + cells and high expression of lectin-2 in malignant plasma cells directly affect TIGIT blockade [167]. Several single and combined checkpoint treatments against TIGIT have been attempted, including TIGIT blockade alone and combined with anti-PD-1 antibody for solid tumors (NCT03119428 and NCT03628677), or combined with anti-PD-L1 antibody treatment in non-small cell lung cancer (NCT03563716). A phase1/2 trial (NCT04150965) of MBS-986207 (anti-TIGIT) as a single agent or in combination with pomalidimide and dexamethasone is being conducted in RRMM.

NKG2A can express on both T cells and NK cells [168]. Monalizumab is a humanised anti-NKG2A antibody that, when combined with a PD-1/PD-L1 axis blocker, exerts a tumour cell killing effect by enhancing NK cell activity and rescues CD8 + T cell function. Current studies include the use of NKG2A blockers in combination with tumour-targeting antibody therapy for the treatment of head and neck squamous cell carcinoma in a phase II clinical trial (NCT02643550); the use of a single blocker of NKG2A for the treatment of gynaecological malignancies (NCT02459301); and the use of Ibrutinib (a BTK inhibitor) in combination with chronic lymphocytic leukemia (CLL) (NCT02557516). In addition, CD96 may also be an immunotherapeutic target against NK cells.

Therefore, we can selectively choose corresponding antibodies for treatment according to the different expression levels in different cells of MM patients to improve the specificity and efficacy of treatment. We hope that there will be new breakthroughs in MM-related immune checkpoint therapies. In solid tumours, we find that the perspective of precision therapy has made corresponding progress in recent years, but has not received corresponding attention and experimental progress in MM. We hope that more researchers will pay attention to the precision therapy of MM and continue to improve its related therapeutic options.

Clinical applications based on immune checkpoint inhibitors are crucial in the immunotherapy of MM and play an important function in immunopathogenesis. However, unfortunately, clinical data on this component are still missing. We consider that an important reason for this phenomenon stems from the slow pace of development and updating of this part of the drug repertoire and its low clinical use. There are a number of immune checkpoints known to affect MM, but the approved use of drugs is still limited to PD-1/PD-L1, etc., so there is correspondingly little clinical data on this component. Many of the advances in several new combinatorial immunological strategies we propose to target immune checkpoints are still limited to solid tumours, and many studies in MM are still in the experimental phase. We will also continue to follow the experimental and research progress in this area to enrich and recognise our understanding in this area and hopefully lead to better clinical and prognostic benefits for more patients.

## Conclusion

In recent years, tremendous progress has been made in immunotherapeutic regimens for MM. Although the emergence of many new drugs has largely prolonged the survival time of patients, it is undeniable that MM remains an incurable malignant myeloproliferative disease. The development of immune checkpoint inhibitors has provided new options for immunotherapy of MM, but the ensuing adverse drug reactions have become a major obstacle to the clinical application of the drugs. Therefore, we summarised the aberrantly expressed immune checkpoints in MM and their mechanisms. We believe that, in addition to the direct use of immune checkpoint inhibitors, is it possible to achieve the effect of reducing the side effects and improving the efficacy of drugs by altering the expression of immune checkpoints or combining them with other drugs. Meanwhile, for the expression differences of immune checkpoints on different cells, we summarise the corresponding ideas of precise treatment, and hope that more relevant clinical trials can explore its feasibility and safety, so that ICI can be applied to clinical treatment as soon as possible and the survival rate of MM patients can be improved.

## Data Availability

Not applicable to this article as no new data were created or analyzed in this study.
